# NAMPT and NAPRT serum levels predict response to anti-TNF therapy in inflammatory bowel disease

**DOI:** 10.3389/fmed.2023.1116862

**Published:** 2023-02-01

**Authors:** Giorgia Colombo, Gian Paolo Caviglia, Alberto Ravera, Elisa Tribocco, Simone Frara, Chiara Rosso, Cristina Travelli, Armando A. Genazzani, Davide Giuseppe Ribaldone

**Affiliations:** ^1^Department of Pharmaceutical Sciences, Università del Piemonte Orientale, Novara, Italy; ^2^Division of Gastroenterology, Department of Medical Sciences, Università di Torino, Turin, Italy; ^3^Department of Drug Sciences, Università degli Studi di Pavia, Pavia, Italy

**Keywords:** NAMPT, NAPRT, inflammatory bowel diseases, anti-TNF, anti-integrins

## Abstract

**Background:**

Nicotinamide phosphoribosyltransferase (NAMPT) and nicotinic acid phosphoribosyl transferase (NAPRT) are key intracellular enzymes that participate in the biosynthesis on NAD but have also been shown to be released as proinflammatory cytokines. A number of reports have shown that circulating NAMPT is increased in serum of patients with inflammatory disorders, including inflammatory bowel diseases (IBD), while nothing is known regarding circulating NAPRT and the presence of both cytokines in IBD patient stools. In the present study, we evaluated eNAMPT and eNAPRT levels in a large cohort of IBD patients not on biological therapy and in a subset that then was prescribed biologics.

**Methods:**

We conducted a retro-perspective study on 180 patients, of which 111 underwent subsequent biological treatment (adalimumab, vedolizumab, and ustekinumab). We analyzed eNAMPT and eNAPRT concentrations in serum and faces of IBD patients, correlating them with response to biologics.

**Results:**

We now report that eNAMPT and eNAPRT are significantly increased in both serum and stools of IBD patients. NAMPT and NAPRT levels correlate with disease severity, with C reactive protein and with serum IL-6 levels. Importantly, levels of NAMPT in patients starting treatment with adalimumab correlate with response failure at three months: patients with levels above 4 ng/ml were significantly less likely to obtain benefit. Serum NAMPT as a biomarker of response yields a sensitivity of 91% and a specificity of 100%.

**Conclusion:**

The present work strongly suggests that a prospective trial evaluating eNAMPT and eNAPRT levels in relation to response to biologicals in IBD should be initiated.

## 1. Introduction

Nicotinamide phosphoribosyltransferase (NAMPT) and nicotinic acid phosphoribosyl transferase (NAPRT) are key intracellular enzymes that participate in the biosynthesis on NAD (1). These cytosolic enzymes have been postulated to represent pharmacological targets in cancer and in immune-mediated disorders as their inhibition leads to depletion of the energetic supply of cancerous and immune cells (2–6).

Nicotinamide phosphoribosyltransferase has long been known to be released by cells (the extracellular form is nowadays referred to eNAMPT but was in the past also referred to as visfatin or PBEF). Initial reports suggested that eNAMPT was an adipokine, i.e., released solely by adipose tissue, but it is now clear that many other cell types also participate in the release of this protein. In the extracellular space, it then exerts a pro-inflammatory cytokine activity (7–10). More recently, the cognate enzyme eNAPRT has similarly been shown to be released by cells (eNAPRT) and to enhance inflammatory responses, although it is unclear if the two proteins share the same receptors and mechanisms (7–10). Upon binding, both cytokines activate in a receptor-mediated manner intracellular pathways including NF-κB and JAK/STAT (9). It has been postulated that both proteins act on TLR4 [(11, 12)], although we have recently shown, at least in myeloid cells, that eNAMPT-mediated synergism with IFNγ is independent of TLR4 (10), prompting the idea that also other receptors may be involved.

Inflammatory bowel diseases (IBD) are a group of chronic inflammatory diseases (of which Crohn’s disease, CD, and ulcerative colitis, UC are the best known entities) whose etiology is not fully established, although genetic predisposition, environmental and dietary factors, alterations of the intestinal microbiome, increase of intestinal permeability, and a deficit of the innate immune response with excessive activation of the T-cell-mediated adaptive immune response are most likely involved (13). In these settings, a number of Authors have shown that serum eNAMPT levels are elevated (14–21). Briefly, serum eNAMPT is increased both in CD and in UC and it is likely to be correlated with the disease stage. Nothing is instead known for eNAPRT or for the presence of either of these proteins in patient stools. Indeed, there are only two reports investigating eNAPRT levels: a report showing an increase in sepsis (9) and a report showing an increase in non-alcoholic fatty liver disease showing a significant increase (22). In particular, in this latter study, eNAPRT, unlike eNAMPT, was associated with advanced fibrosis which was similarly distributed across fibrosis stages.

The involvement of eNAMPT in IBD has recently also been confirmed using murine animal models. Briefly, chemically induced colitis is characterized by high levels of serum eNAMPT (20) and eNAPRT and the reduction of eNAMPT levels *via* a neutralizing antibody significantly ameliorates the symptoms and the associated inflammation (20).

More than 6 million people are affected by IBD globally (23) and the number of pharmacological alternatives for those that require more aggressive treatments are fortunately growing (and consequently also the costs for health systems). Yet, biomarkers able to guide drug choice have been largely elusive (24). Yet, these would be fundamental to improve patient care avoiding a trial-and-error therapeutic approach. In a set of 3 separate small cohorts of IBD patients we have previously suggested that serum eNAMPT levels could have a prognostic value on anti-TNF response (20). In the present study, we replicated these findings on a larger cohort of patients treated with adalimumab and also evaluated eNAMPT and eNAPRT levels in a large cohort of IBD patients not on biological therapy. We now report that eNAMPT and eNAPRT levels are elevated in IBD patients in both blood and faces, that their levels correlate with pathological score and with high sensitivity C reactive protein (hsCRP), and, most importantly, confirm that they represent predictive biomarkers for response to anti-TNF treatment.

## 2. Materials and methods

### 2.1. Patients

The study was approved by AOU Città della Salute e della Scienza di Torino–A. O. Mauriziano–A.S.L. TO1 Ethical Committee (n. 0056924 of 08/06/2016). Patients referring to the local IBD center with an IBD diagnosis according to ECCO criteria (25, 26) not yet progressed to biologic therapy were consecutively recruited (*n* = 180) and a blood sample was withdrawn upon informed consent. If available, a stool sample (*n* = 62) was also taken. In patients (*n* = 111) in which clinical judgment suggested that a biologic therapy should have been initiated (adalimumab *n* = 62; vedolizumab *n* = 40; ustekinumab *n* = 9), a blood sample was also taken after 3 months from the first administration of the biologic. Choice of the biologic to be prescribed was done as for clinical judgment. Patient characteristics, schedule of drug therapy upon referral and maintenance are summarized in Table 1. Clinical response to biologic therapy was defined as a decrease in the Harvey-Bradshaw index (HBI) greater than or equal to 3 (or HBI ≤ 4) or in the partial Mayo (pMAYO) score greater than or equal to 2 (or pMAYO ≤ 1), in the absence of corticosteroid therapy (26). Patients who discontinued biologic treatment, or those lost to follow-up, were considered as cases of treatment failure (intention to treat analysis). Controls (*n* = 22) were represented by healthcare or laboratory personnel with no history of IBD (*n* = 17) and by patients with irritable bowel syndrome (*n* = 5).

**TABLE 1:** Baseline clinical characteristics of the study population.

Characteristics	IBD	CD	UC
Number of patients	180	128	52
Median age (range)	45 (17–80)	43 (17–80)	51 (19–78)
Sex (M/F)	110/70	78/50	31/20
BMI	23.78 (14.45–51.0)	24.07 (14.45–34.30)	22.95 (16.98–51.0)
**Montreal Classification**			
(CD: L1/L2/L3/L4); UC (E1/E2/E3)	–	41/10/69/3*	6/18/26*
Clinical activity (mean, 95% CI) (CD: HBI; UC: pMAYO)	–	6.2 (5.4–7.0)	3.7 (2.9–4.5)
Median Years of illness (range)	11 (1–49)	12 (1–49)	5,5 (2–38)
Remission/mild/moderate/severe	57/47/64/12	46/38/40/4	11/9/24/8
Previous surgery for IBD (yes/no)	71/109	62/66	8/44
Smoke (current/never/former)	39/75/66	32/53/43	7/21/24
**Biochemical activity**			
FC (ug/g) median (95% CI)	577 (366–872)	554 (332–872)	579 (162–1,800)
hsCRP (mg/l) median (95% CI)	6.0 (4.0–7.9)	5.7 (4.0–7.9)	6.8 (3.3–12.8)
ESR (± /NA)	71/67/42	52/49/27	14/21/17
Systemic corticosteroids (yes/no)	74/106 (41.1%)	49/79 (38.2%)	27/25 (51.9%)

*For some patients (5 for CD and 2 for UC) the Montreal classification was not available.

### 2.2. Stool processing and sample preparation

Stools were stored at −80°C, thawed and weighted before processing. Faces were homogenized in Lysis Buffer (20 mM HEPES, 100 mM NaCl, 5 mM EDTA, 1% Non-idet-P40 + Protease and Phosphatase Inhibitor Cocktail, Sigma) and centrifuged at 12,000 rpm for 10′ to discard debris. 100 μl of supernatants were used for ELISA assay.

### 2.3. NAMPT and NAPRT determination by ELISA

Serum eNAMPT and faucal NAMPT were evaluated with a commercially available sandwich enzyme-linked immunosorbent assay for human NAMPT (ELISA kit from AdipoGen Inc., Seoul Korea). Serum eNAPRT and faucal NAPRT were evaluated with a commercially available sandwich enzyme-linked immunosorbent assay for human NAPRT (ELISA kit from Abbexa Ltd., Cambridge, UK). Faucal NAMPT and NAPRT amount were normalized on the weight of the homogenized sample (ng/μg of samples).

### 2.4. Measurement of serum cytokines

A panel of cytokines including IL-6, IL-8, IL-10, TNFα, TGFβ, and IL-33 was measured in serum samples by Multiplex Immunoassay (Bio-Plex^®^, Bio-Rad Laboratories, Pleasanton, CA, USA) on the Luminex^®^ 200 system (Luminex Corporation, Austin, TX, USA) according to manufacturers’ instruction. For each cytokine, an individual standard curve was generated, and the results were given in pg/mL.

### 2.5. Statistics

This was an exploratory study and therefore no formal statistical plan was pre-planned. Data are presented as mean ± SEM in Table 1 compare 95% CI or median and range. The normality of data distributions was evaluated using the Shapiro–Wilk test. Parametric (unpaired *t*-test and One-way analysis of variance (ANOVA) followed by Tukey’s *post hoc*) or non-parametric (Mann–Whitney U test and One-way Kruskal–Wallis H test followed by Dunn’s *post hoc*) statistical analysis were used. Receiver operating characteristic (ROC) curve analysis was used to test the ability of eNAMPT and eNAPRT to discriminate between patients who are responsive or not to the biological drugs. Diagnostic accuracy is reported as area under the curve (AUC) value. Pearson’s correlation and Multivariate Cox proportional hazard analysis were also performed. All statistical assessments were two-sided and a value of *P* < 0.05 was considered statistically significant. Statistical analysis was performed using GraphPad Prism software (GraphPad Software, Inc., San Diego, CA, USA).

## 3. Results

### 3.1. Serum and faucal NAMPT and NAPRT levels are increased in IBD patients

**FIGURE 1:**
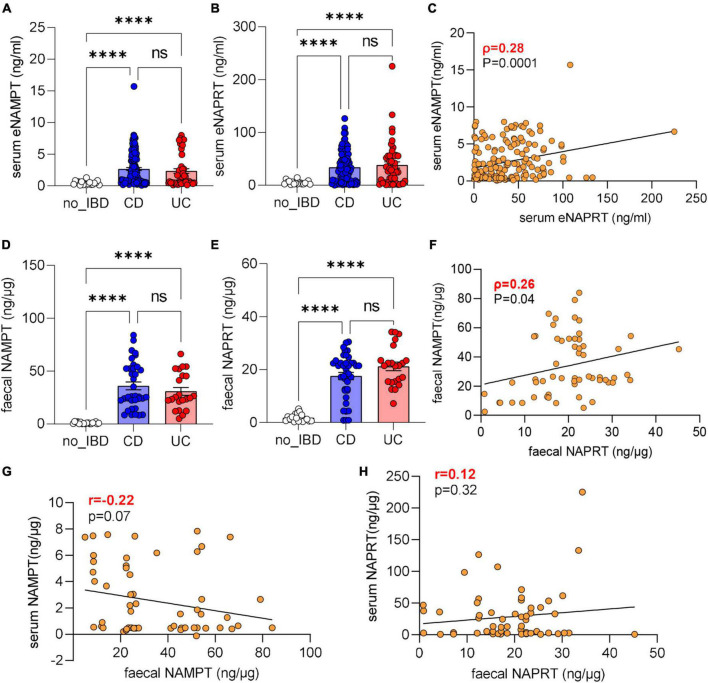
eNAMPT and eNAPRT are increased in serum and faces of IBD patients. **(A)** Serum eNAMPT levels and **(B)** eNAPRT levels (healthy controls *N* = 18: CD *n* = 128; UC = 52). **(C)** Pearson correlation between serum eNAMPT and eNAPRT levels in IBD patients. **(D)** Faucal NAMPT and **(E)** NAPRT levels (healthy controls *N* = 18: CD *n* = 40; UC = 22). **(F)** Pearson correlation between faucal NAMPT and faucal NAPRT levels in IBD patients. **(G)** Pearson correlation between faucal NAMPT and serum eNAMPT levels in IBD patients. **(H)** Pearson correlation between faucal NAPRT and serum eNAPRT levels in IBD patients. *P*-value: ^****^p < 0.0001.

We first determined eNAMPT and eNAPRT levels in patients which were naïve to biologics. We found that that eNAMPT levels were increased in IBD patients (median 1.53 ng/ml) compared to a healthy cohort (median 0.36 ng/ml; median for IBS patients included in this cohort 0.42 ng/ml). There were no statistical differences between the entire IBD population, UC (median 1.80 ng/ml) or CD patients (median 1.28 ng/ml, Figure 1A) which were all statistically higher than controls. eNAPRT levels were higher compared to eNAMPT at baseline in healthy patients (median 4.32 ng/ml; median for IBS patients 2.33 ng/ml) and increased approximately seven-fold in the entire IBD population (median 28.47 ng/ml), as well as in UC (median 30.15 ng/ml) and CD (median 28.22 ng/ml) patients (Figure 1B). For both cytokines, there was no difference between male (median eNAMPT 1.25 ng/ml, eNAPRT 28.16 ng/ml) and female (median eNAMPT 1.77 ng/ml, eNAPRT 29.2 ng/ml) patients. When comparing intra-patient levels, there was a poor correlation between these two cytokines (Figure 1C), while we observed a difference of eNAMPT and eNAPRT levels between patients with active and remittent disease (eNAMPT median UC_active 1.65 ng/ml vs. UC_remission 0.48 ng/ml; CD_active 2.20 ng/ml vs. CD_remission 1.23 ng/ml; eNAPRT median UC_active 34.53 ng/ml vs. UC_remission 5.38 ng/ml; CD_active 33.74 ng/ml vs. CD_remission 18.28 ng/ml). There was no correlation of either eNAMPT or eNAPRT levels with age (*p* = 0.82 and 0.88, respectively) or years from diagnosis (*p* = 0.40 and *p* = 0.50, respectively). Instead, eNAMPT was positively correlated with BMI, as reported by others (23, 24), while we did not find any correlation with eNAPRT.

We also analyzed the levels of these cytokines in faces with similar results, and in this instance the levels of eNAMPT and eNAPRT were comparable between them. Briefly, faucal eNAMPT and faucal eNAPRT were elevated in IBD patients (median 25.55 and 18.87 ng/μg, respectively) compared to healthy subjects (median 0.81 and 1.25 ng/μg; median for the IBS patients included 1.22 and 1.25 ng/μg). Again, there were no differences (Figures 1D, E) between UC (eNAMPT median 24.96 ng/μg and eNAPRT median 21.45 ng/μg) and CD patients (eNAMPT median 25.82 ng/μg and eNAPRT median 20.13 ng/μg). In analogy to what found in serum, there was no correlation between faucal NAMPT and faucal NAPRT levels (Figure 1F). We also performed Pearson’s correlation between serum eNAMPT and eNAPRT with faucal eNAMPT and eNAPRT, respectively, but also in this case, no correlation was found (Figures 1G, H).

### 3.2. Serum and faucal eNAMPT and eNAPRT are positively correlated with hsCRP and the severity score

**FIGURE 2:**
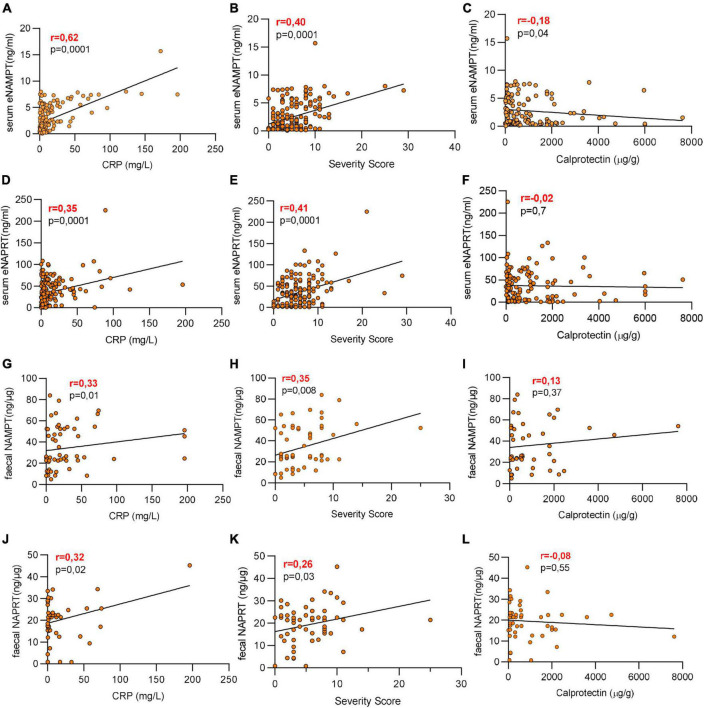
Serum and faucal eNAMPT and eNAPRT are positively correlated with hsCRP and the pathological score. **(A–F)** Pearson correlation between serum eNAMPT or eNAPRT and **(A,D)** hsCRP levels, **(B,E)** pathological score and **(C,F)** calprotectin in IBD patients (healthy controls *N* = 18: CD *n* = 128; UC = 52). **(G–L)** Pearson correlation between faucal NAMPT or eNAPRT and **(G,J)** hsCRP levels, **(H,K)** pathological score and **(I,L)** calprotectin in IBD patients (healthy controls *N* = 18: CD *n* = 40; UC = 22).

We next analyzed the relationship between eNAMPT and eNAPRT serum levels and the baseline clinical features. As shown in Figures 2A–C, serum eNAMPT was positively and significantly correlated with hsCRP (*p* = 0.0001; *r* = 0.62) and the pathological score (*p* = 0.0001; *r* = 0.40), resembling the inflammatory condition of patients, but did not correlate with faucal calprotectin. Moreover, also serum eNAPRT levels were positively correlated with hsCRP (*p* = 0.001; *r* = 0.35) and the pathological score (*p* = 0.0001; *r* = 0.41) with no correlation with calprotectin (Figures 2D–F). The same analysis was performed with the faucal values. For faucal eNAMPT, we identified a slight positive correlation with hsCRP (*p* = 0.01; *r* = 0.30, Figure 2G) and the pathological score (*p* = 0.008; *r* = 0.33, Figure 2H), again with no correlation with calprotectin (Figure 2I). This was paralleled also by faucal eNAPRT that slightly correlated with hsCRP (*p* = 0.02; *r* = 0.32, Figure 2K) and the pathological score (*p* = 0.003; *r* = 0.28, Figure 2L), but not with calprotectin (*r* = −0.08, Figure 2J).

Last, we evaluated the correlation between eNAMPT or eNAPRT serum levels and IL-6, IL-8, IL-10, TNFα, TGFβ, and IL-33. As depicted in Table 2, a significant positive correlation was found with IL-6 and a negative correlation was found with IL-10 for both eNAMPT and eNAPRT, with no other association found.

**TABLE 2:** Correlation between eNAMPT and eNAPRT levels with inflammatory cytokines.

Parameter	Serum eNAMPT (ng/ml)	Serum eNAPRT (ng/ml)
Age	*r* = 0.01; *p* = 0.82	*r* = 0.01; *p* = 0.88
Sex (M/F)	*r* = −0.1; *p* = 0.04	*r* = −0.1; *p* = 0.04
Years of illness	*r* = 0.01; *p* = 0.4	*r* = 0.01; *p* = 0.5
BMI	*r* = 0.56; *p* = 0.001	*r* = 0.03; *p* = 0.1
IL-6 (pg/ml)	*r* = 0.36; *p* = 0.001	*r* = 0.45; *p* = 0.001
IL-8 (pg/ml)	*r* = 0.014; *p* = 0.81	*r* = 0.03; *p* = 0.74
IL-10 (pg/ml)	*r* = −0.55; *p* = 0.001	*r* = −0.42; *p* = 0.001
TNFα (pg/ml)	*r* = −0.03; *p* = 0.04	*r* = 0.20; *p* = 0.03
TGFβ1 (pg/ml)	*r* = 0.04; *p* = 0.5	*r* = 0.17; *p* = 0.18
IL-33 (pg/ml)	*r* = 0.17; *p* = 0.11	*r* = 0.12; *p* = 0.28

### 3.3. Serum eNAMPT levels are predictors of anti-TNF response

We then proceeded to analyses eNAMPT and eNAPRT levels in patients who underwent subsequent biologic treatment.

**FIGURE 3:**
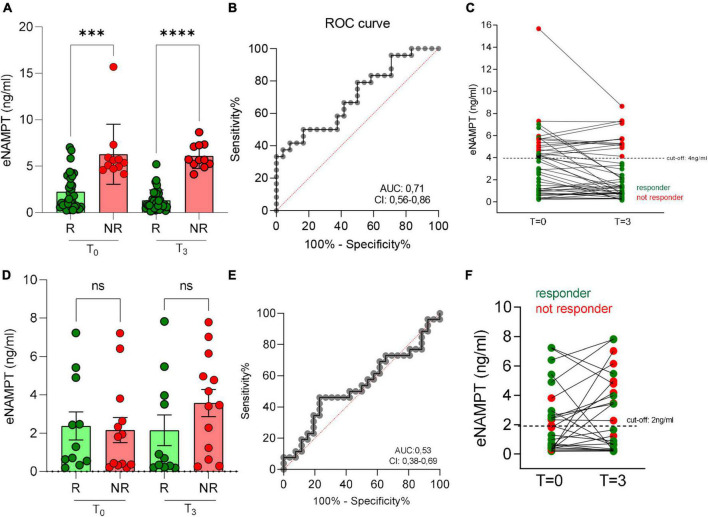
Serum eNAMPT levels and response to anti-TNF treatment in IBD patients. **(A)** eNAMPT levels in responders (R) and non-responders (NR) to adalimumab at baseline (*T* = 0) and after 3 months from the first infusion (*T* = 3). *N* = 62: CD = 58, UC = 4. **(B)** ROC curve of adalimumab response. **(C)** eNAMPT levels in single patients after adalimumab infusion. Red dots represent NR, green dots represent R. *N* = 62: CD = 58, UC = 4. **(D)** eNAMPT levels in R and NR to vedolizumab at baseline (*T* = 0) and after 3 months from the first infusion (*T* = 3). *N* = 40: CD = 23, UC = 17. **(E)** ROC curve of vedolizumab response. **(F)** eNAMPT levels in single patients after vedolizumab infusion. *N* = 40: CD = 23, UC = 17. Red dots represent NR, green dots represent R. *P*-value: ^***^p < 0.001 and ^****^p < 0.0001.

For those that were administered with adalimumab, median levels of eNAMPT decreased, albeit not significantly, in responsive patients but not in non-responsive patients after 3 months of treatments (Figure 3A). More importantly, though, we noticed a significant difference in eNAMPT basal levels between responsive and non-responsive patients, as defined in the methods section, observing lower levels in responsive (T0 median = 1.34 ng/ml) compared to non-responsive patients (T0 median = 5.36 ng/ml) (Figure 3A). Through ROC analysis (AUC 0.71, Figure 3B), we determined 4 ng/ml as the cut-off of eNAMPT levels. As shown in Figure 3C, in the group with eNAMPT levels over the cut-off only a minority of patients responded (2/13; 15%), while all patients with eNAMPT levels below the cut-off responded to anti-TNF treatment (49/49; 100%). It is well known that eNAMPT correlates with BMI and therefore we investigated whether BMI could also predict response to adalimumab in our patients, although the correlation between BMI and response to biologics has been investigated previously with contradictory results (25). In our cohort, BMI did not correlate with response.

We also evaluated eNAMPT in 40 patients in treatment with vedolizumab at baseline and after 3 months. In this case, no correlation between eNAMPT levels and response to treatment was observed (Figures 3D–F). Last, we evaluated eNAMPT levels in a small cohort (*N* = 9) of patients treated with the anti-IL-12/IL-23 drug, ustekinumab. Ustekinumab in responsive patients reduced the levels of eNAMPT and the difference of eNAMPT between responsive and not responsive patients was significant (Supplementary Figure 1A). Calculating the cut-off through ROC analysis (Supplementary Figure 1B), no patient with eNAMPT levels above 4 ng/ml (Supplementary Figure 1C) responded to treatment (0/5), while all patients with eNAMPT levels below 4 ng/ml responded (4/4).

### 3.4. Serum eNAPRT levels are predictors of anti-TNF response

**FIGURE 4:**
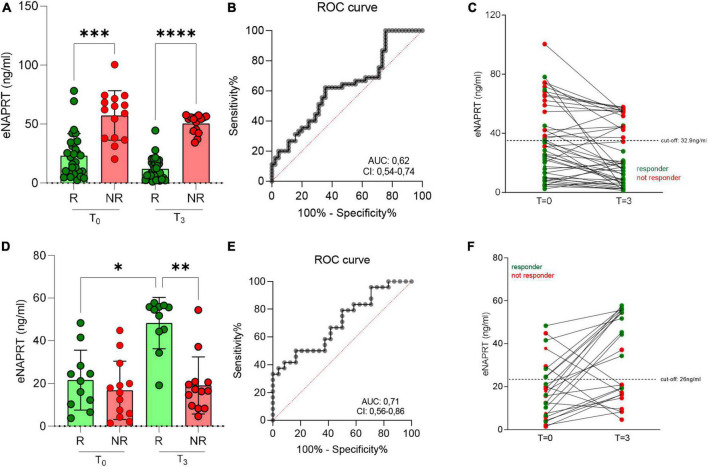
Serum eNAPRT levels and response to anti-TNF treatment in IBD patients. **(A)** eNAPRT levels in responders (R) and non-responders (NR) to adalimumab at baseline (*T* = 0) and after 3 months from the first infusion (*T* = 3). *N* = 62: CD = 58, UC = 4. **(B)** ROC curve of adalimumab response. **(C)** eNAPRT levels in single patients after adalimumab infusion. Red dots represent NR, green dots represent R. *N* = 62: CD = 58, UC = 4. **(D)** eNAPRT levels in R and NR to vedolizumab at baseline (*T* = 0) and after 3 months from the first infusion (*T* = 3). *N* = 40: CD = 23, UC = 17. **(E)** ROC curve of vedolizumab response. **(F)** eNAPRT levels in single patients after vedolizumab infusion. *N* = 40: CD = 23, UC = 17. Red dots represent NR, green dots represent R. *P*-value: *p < 0.05; ^**^p < 0.01; ^***^p < 0.001, and^****^p < 0.0001.

We next performed the same analysis correlating eNAPRT levels with response to adalimumab. Again, a significant difference in basal eNAPRT levels between responsive and not responsive patients could be observed. Briefly, responsive patients showed lower levels (median = 19.8 ng/ml) compared to non-responsive patients (median = 66.2 ng/ml; Figure 4A). The cut-off of 33 ng/ml, determined by ROC analysis (Figure 4B) was able to discriminate patients less likely to respond to anti-TNF treatment (3/13; values above) from those more likely to respond (39/42; values below; Figure 4C).

No correlation was instead found in patients that were treated with vedolizumab (Figures 4D–F) or ustekinumab (Supplementary Figure 2).

We also performed a multivariate analysis considering eNAMPT, eNAPRT, hsCRP and calprotectin levels. As seen in Table 3, baseline eNAMPT and eNAPRT levels are independent predictive factors, while, as already known (25), neither baseline hsCRP nor baseline calprotectin predicted response to anti-TNF therapy, albeit their levels are obviously of clinical usefulness to follow drug responses (27).

**TABLE 3:** Multivariate Cox proportional hazard analysis examining factors associated with treatment success in ADALIMUMAB-treated patients with IBD.

Variables	Hazard Ratio	95% CI	*P*-value
NAMPT	1.7555	0.5062 to 0.985	0.0441
NAPRT	2.9468	0.8955 to 0.9967	0.0417
Calprotectin	1.002	0.9977 to 1.005	0.4097
hsCRP	1.004	0.9882 to 1.018	0.5975

## 4. Discussion and conclusion

In the present exploratory and confirmatory study, we show that (i) eNAMPT is elevated in serum and faces of IBD patients; (ii) eNAPRT, its cognate enzyme, is similarly increased; (iii) eNAMPT and eNAPRT are predictors of response to adalimumab.

Unlike the increased levels of faucal eNAMPT and serum and faucal eNAPRT in IBD, which are reported here for the first time, the levels of eNAMPT in serum of IBD patients had been investigated previously. Our data is in line previous reports (Supplementary Table 1 for a synopsis of the previously published articles) showing an elevation of eNAMPT in unselected patients and with most of the studies which show a correlation with disease severity. Unfortunately, despite the number of previous investigations, the heterogeneity of clinical protocols (Supplementary Table 2) does not allow to meta-analyses the data, although we believe the elevation in serum eNAMPT is now firmly confirmed. Similarly, our study focused on adalimumab, and while small trials for other treatments (e.g., corticosteroids, azathioprine, other anti-TNF agents) are present in the literature (Supplementary Table 1), larger trials should be performed to inform clinicians.

Our data also support the previous observations that eNAMPT does not correlate with age and years of disease, while our correlation with hsCRP is supported by Saadoun et al. (21), although other Authors failed to find this correlation. Our data is instead in line with Neubauer showing a correlation with IL-6 while we are the first to investigate and report a negative correlation with IL-10. The link between IL-6 and eNAMPT is biologically plausible and it has been shown that in mice eNAMPT induces the transcription of IL-6 in the small intestine and leads to an increase in circulating IL-6 (14). For these correlations, further studies are required to firmly establish the presence of a link. It should be noted that it has been previously reported that TNFa levels do not correlate to response to anti-TNF therapy (27) and therefore the lack of association between this cytokine and eNAMPT/eNAPRT is in line with this finding.

In the present investigation, we also looked at eNAPRT, an enzyme involved in NAD metabolism, which is structurally similar to eNAMPT and that has only recently been shown to be released and act as a cytokine (9). Our data suggests that eNAPRT is also elevated in IBD and correlates with the same factors as eNAMPT. Surprisingly, though, eNAMPT and eNAPRT levels in serum only show a lax correlation between them, suggesting a different involvement and regulation. This finding is supported by a recent report on non-alcoholic fatty liver disease, in which these two cytokines were correlated to different factors (22). Such data is further strengthened by the observations in faces. We show, for the first time, that both cytokines are elevated in faces of IBD patients, but their relative ratio compared to serum is different, with higher concentrations of eNAPRT in serum and higher concentrations of eNAMPT in faces.

The source of eNAMPT and eNAPRT in blood is at present unknown, and it is likely to derive from immune cells. For example, myeloid cells are an important source for eNAMPT, as reported in several manuscripts (10, 14, 28). For example, myeloid cells are an important source for eNAMPT, as reported in several manuscripts (10, 14, 28). On the other hand, eNAMPT and eNAPRT are also known to be released by the adipose tissue, which could be a further contributor. Regarding the presence of eNAMPT and eNAPRT in faces, there have been no previous reports, and it is possible that the source may be similar to that of calprotectin, i.e., myeloid cells (29). Overall, eNAMPT and eNAPRT levels both in serum and faces may be seen as inflammatory biomarkers, not superimposable to calprotectin or hsCRP, or may define a different disease entity.

Most importantly, in the present study we evaluated whether these cytokines had any predictive role for biological therapy and performed a confirmative trial of our previous results that suggested that eNAMPT levels in active disease predicted response to anti-TNF therapy (infliximab in both pediatric and adult patients and adalimumab in adult patients). Briefly, we have previously shown, using three separate small cohorts for a total of 79 patients, that all patients with low eNAMPT levels (below 4.5 ng/ml) responded to therapy at 3 months while only half of the patients with higher eNAMPT levels responded (20). The data in the present manuscript is perfectly in line with the previous data both in terms of effects and of ROC curves. Cumulating the data presented here with the data presented previously and using 4 mg/ml as the threshold, the prognostic effect of eNAMPT yields a sensitivity of 91% and a specificity of 100% for response to anti-TNF. eNAPRT levels would also appear predictive, although it does not have a replication cohort as it was not performed previously, and at present pooling data from eNAMPT and eNAPRT levels does not appear to improve the sensitivity. We have also evaluated the Positive predictive value (PPV) that gives us the proportion of cases giving positive test results who are already patients, this value is of 97%. External support for the hypothesis that eNAMPT may be a true predictive biomarker comes from the report that high expression of *Nampt* mRNA associated to a lack of response to golimumab, yet another anti-TNF agent (30).

Preliminary data would also suggest that neither eNAMPT nor eNAPRT are able to predict responses to ustekinumab, while the data on vedolizumab is too preliminary to draw conclusions.

The present study should be read in light of the following limitations: (i) all patients were recruited from a single clinical center, although this is mitigated by the fact that it replicates three smaller studies performed in different centers with two separate anti-TNF drugs; (ii) the study was single-arm and therefore there is the risk of selection bias of the patients enrolled to the different biological agents; (iii) the study design was retrospective and exploratory, with no primary outcome defined *a priori*, although blood was collected prospectively, as were clinical and biochemical parameters; (iv) response to therapy was assessed clinically, but since response was assessed at 3 months, it would have been unethical to have a colonoscopy down to the end of the induction phase in all patients; (v) the data obtained on patients on ustekinumab should be only viewed as descriptive given the low number of patients (*n* = 9) while the data on vedolizumab should be viewed as exploratory (*n* = 40) due to lack of power; (vi) we did not correlate eNAMPT/eNAPRT levels with complete blood count or with C-reactive protein to albumin ratio, as in local clinical protocols these are not performed in outpatients.

In light of our results that clearly suggest that eNAMPT can be used as a humoral marker to direct first line treatment choice of the fact that this result was replicated and of the limitations of the study, the present work strongly suggests that a prospective trial testing the possibility that eNAMPT and eNAPRT levels may inform on first-line biologic treatment in IBD should be initiated.

## Data availability statement

The raw data supporting the conclusions of this article will be made available by the authors, without undue reservation.

## Ethics statement

The studies involving human participants were reviewed and approved by The study protocol is compliant with the ethics guidelines of the 1975 Declaration of Helsinki and was approved by AOU Città della Salute e della Scienza di Torino–A. O. Mauriziano–A.S.L. TO1 Ethical Committee (n. 0056924 of 08/06/2016). This committee confirmed that no formal written consent for ethics approval was required in this study. Data were anonymised after linkage between databases. The patients/participants provided their written informed consent to participate in this study.

## Author contributions

GC and AG designed protect study. DR, AR, SF, and ET collected clinical data. GC did the experiments. GPC stocked samples. GPC, CR, and ET performed BioPlex analysis. GC, CT, AG, and DR discussed and interpreted findings. AG, GPC, DR, and AG wrote the manuscript. All of the authors have seen and approved the final version of the manuscript.
