# Supplementary material for: NAMPT and NAPRT serum levels predict response to anti-TNF therapy in inflammatory bowel disease
Source: Front Med (Lausanne). 2023 Feb 1;10:1116862. doi: 10.3389/fmed.2023.1116862 (PMC9928959; doi:10.3389/fmed.2023.1116862)
Supplement: Supplementary file 1 [file Data_Sheet_1.pdf]

Supplementary Material

NAMPT and NAPRT serum levels predict response to anti-TNF therapy in inflammatory bowel disease

Giorgia Colombo[1§], Gian Paolo Caviglia[2§], Alberto Ravera[2], Elisa Tribocco[2], Simone Frara[2], Chiara Rosso[2], Cristina Travelli[3], Armando A. Genazzani[1]#*, Davide Giuseppe Ribaldone[2]#*

[1]Department of Pharmaceutical Sciences, Università del Piemonte Orientale, Novara, 28100, Italy.

[2]Division of Gastroenterology, Department of Medical Sciences, Università di Torino, 10100, Italy.

[3] Department of Drug Sciences, Università degli Studi di Pavia, Pavia, 27100, Italy.

§ These authors contributed equally to the work

# These authors contributed equally to the work

*** Correspondence:**

Corresponding authors, armando.genazzani@uniupo.it, davidegiuseppe.ribaldone@unito.it

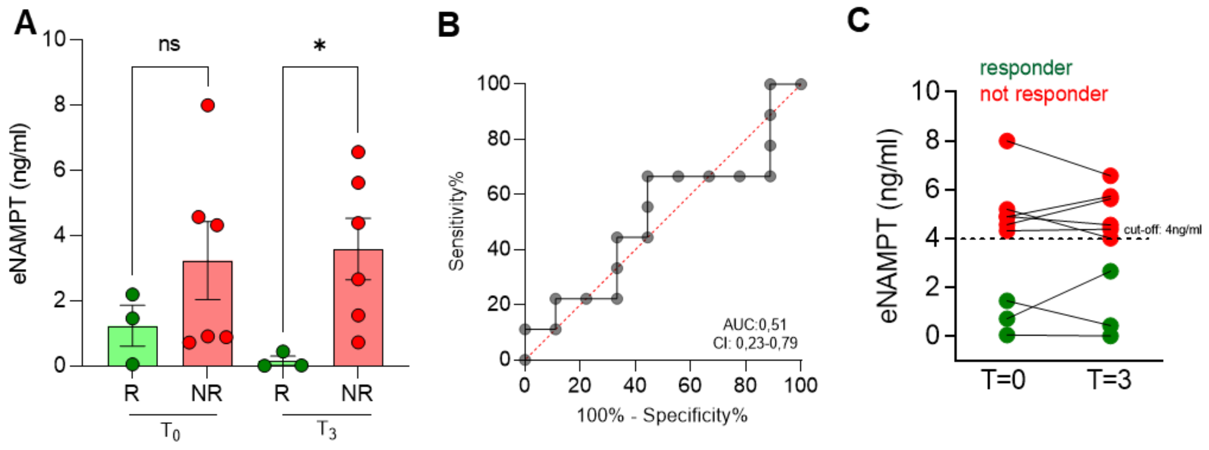

Supplementary Figure 1. (**A**) eNAMPT levels in R and NR to ustekinumab at baseline (T=0) and after 3 months from the first infusion (T=3). N=9: CD=5, UC=4. (**B**) ROC curve of ustekinumab

response. (**C**) eNAMPT levels in single patients after ustekinumab infusion. N=9: CD=5, UC=4. Red dots represent NR, green dots represent R.

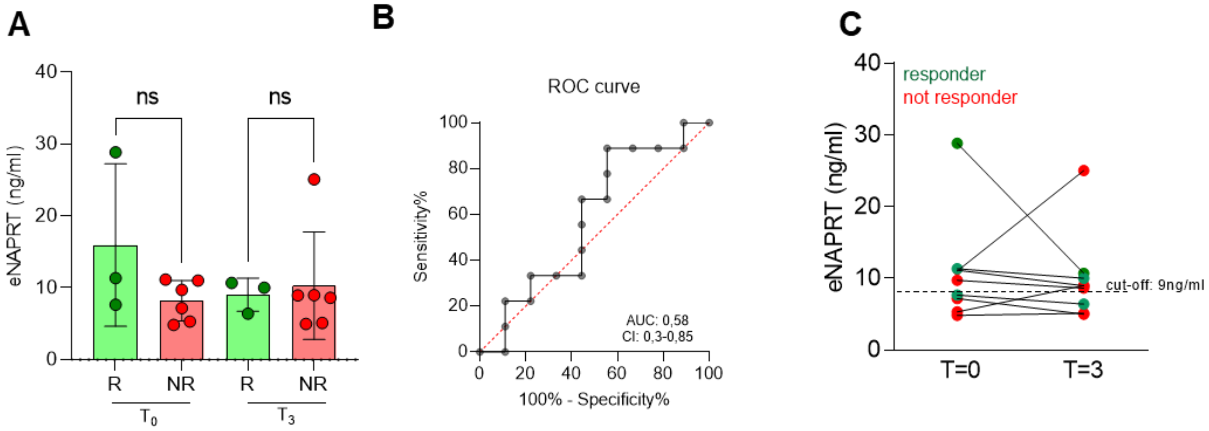

Supplementary Figure 2. (**A**) eNAPRT levels in R and NR to ustekinumab at baseline (T=0) and after 3 months from the first infusion (T=3). N=9: CD=5, UC=4. (**B**) ROC curve of ustekinumab response. (**C**) eNAPRT levels in single patients after ustekinumab infusion. N=9: CD=5, UC=4. Red dots represent NR, green dots represent R.

	Moschen, 2007[13]	Valentini, 2007[19]	Waluga, 2014[15]	Dogan, 2016[14]	Terzoudis, 2016[17]	Neubauer, 2019[16]	Colombo, 2020[12]	Saadoun, 2021[18]	Colombo, present contribution
**Number of Patients**	HC 38<br>CD 39<br>UC 35	HC 37<br>CD 67<br>UC 61	HC 16<br>CD 24<br>UC 16	HC 29<br>CD 0<br>UC 31	HC 98<br>CD 68<br>52 UC	no-IBD 60[#]<br>CD 113<br>UC 127	HC 11<br>CD 64<br>UC 22	HC 30<br>CD 29<br>UC 56	HC 18<br>CD 128<br>UC 52
	**Evaluation**								
eNAMPT<br>(HC vs IBD)[a]	↑	n.r.	↑	n.r.	n.r.	↑	↑	↑	↑
eNAMPT<br>(active *vs* inactive/remission)	0 in CD<br>↑ in UC	0 in CD<br>↑ in UC	n.r.	n.r. in CD<br>↑ in UC	n.r.	0 in CD<br>↑ in UC	n.r.	n.r.	↑ in CD<br>↑ in UC
CRP	n.r.	0	n.r.	n.r.	0	0 (hsCRP)	n.r.	+	+
Severity	n.r.	n.r.	0 Montreal classification	n.r.	n.r.	+ Rachmilewitz index (UC)<br>+ Mayo endoscopic score (UC)	n.r.	+ Montreal classification	+ Severity score (HBI and Mayo's score)
Calprotectin	n.r.	n.r.	n.r.	n.r.	n.r.	n.r.	n.r.	+	0
IL-6	n.r.	n.r.	n.r.	n.r.	n.r.	+	n.r.	n.r.	+
IL-10	n.r.	n.r.	n.r.	n.r.	n.r.	n.r.	n.r.	n.r.	-
IL-8	n.r.	n.r.	n.r.	n.r.	n.r.	+	n.r.	n.r.	0
TNFα	n.r.	n.r.	n.r.	n.r.	n.r.	+ (active disease)	n.r.	n.r.	0
Age	n.r.	n.r.	n.r.	n.r.	n.r.	0	n.r.	0	0
Years of disease	n.r.	0	0	n.r.	0	0	n.r.	n.r.	0

Supplementary Table I. Evidence so far gathered on eNAMPT in IBD patients. Information was extracted either from the text or from the Figures/Tables of the indicated manuscripts. HC healthy controls; CD Crohn's disease; UC ulcerative colitis; n.r. not reported; + positive correlation; 0 no correlation or no change; - negative correlation; ↑ increase; [a]overall population studied, independently of variables (*e.g.,* active, remission, severity).[#]no-IBD included both healthy patients and patients suffering from irritable bowel disease.
